# Apparent clearance of valproic acid in elderly epileptic patients: estimation of the confounding effect of albumin concentration

**DOI:** 10.3109/03009734.2011.640412

**Published:** 2012-02-15

**Authors:** Natalia Lampon, J. Carlos Tutor

**Affiliations:** Unidad Monitorización Fármacos, Laboratorio Central, Hospital Clínico Universitario, Instituto de Investigación Sanitaria (IDIS), 15706 Santiago de Compostela, Spain

**Keywords:** Albumin, apparent clearance, elderly patients, unbound fraction, valproic acid

## Abstract

**Background.:**

Valproic acid (VPA) apparent clearance (CL) estimated from total serum concentrations is analogous in elderly and non-elderly adult patients. As drug–protein binding decreases in old age, the aim of our study was to evaluate the confounding effect of the serum albumin concentration on the VPA apparent CL in elderly patients.

**Methods.:**

In 102 epileptic out-patients treated with VPA in monotherapy, serum total steady-state trough concentrations (Css) were determined. Css concentrations were normalized for a 42 g/L albumin concentration (Css_N_), and the apparent CL and normalized apparent CL_N_ were calculated.

**Results.:**

A poor concordance of 53% was found in the classification of Css and Css_N_ levels of VPA as subtherapeutic, therapeutic, or supratherapeutic dose. In the elderly (≥65 years) and non-elderly adult patients, the VPA apparent CL was similar; however, normalized apparent CL_N_ was significantly lower in older patients (*P* < 0.01), with a 40% median decrease.

**Conclusions.:**

Total VPA concentrations should be interpreted with caution, mainly in older patients, in which determination of unbound or normalized total drug concentrations may be clinically useful. Normalization of total concentrations permits an estimation of the masking effect of serum albumin concentrations on the VPA apparent CL in elderly patients.

## Introduction

Although valproic acid (VPA) was initially approved for the treatment of refractory absence seizures, it is now recognized that it has a broad-spectrum antiepileptic activity (1). Similarly, VPA may be effective in a variety of psychiatric and neurological diseases such as bipolar disorders, schizophrenia, depression, neurological pain, migraine headaches, Alzheimer’s disease, and other neurodegenerative illnesses (2).

VPA is well absorbed from all oral dosage forms, with a bioavailability greater than 90%. The drug, structurally related to free fatty acids, is highly ionized at physiological pH and therefore presents to a high degree bound to plasma proteins, primarily to albumin (3–5). In conditions of hypoalbuminemia (even moderate) there is a significant increase in the free fraction (percent unbound) of the drug; likewise, for total serum concentrations up to 75 μg/mL, VPA is about 90% bound to albumin, but above this concentration binding saturates with an increase of the free fraction (4).

The effect of age on the VPA pharmacokinetics has been widely studied, although there is clearly a need for improved methodology in the assessment of age-related changes in pharmacokinetics (6). The apparent clearance (CL) calculated from total serum VPA concentrations is similar in elderly and non-elderly adult patients (7–10); however, in a clinical context these findings must be interpreted with caution, considering that VPA plasma protein binding decreases in old age, with an accompanying increase of the free fraction of the drug (5,8,11).

For highly protein-bound drugs, such as VPA, diminished plasma protein binding is associated with a decrease of the serum total concentration (protein-bound plus free drug), and consequently with an increased apparent CL; however, it would be misleading to assume that the amount of drug eliminated per unit of time is also increased (12). The lower total concentration of the drug is associated with a lower protein-bound concentration and increased free fraction, although the unbound concentration and the amount of drug eliminated per unit of time remain unchanged. Therefore, the pharmacological effect in these conditions will be analogous to that produced by the higher total drug concentration obtained under normal protein binding conditions for the same daily dose (12).

The decrease of serum albumin concentrations in old age is well established (13), and the main aim of our study was to evaluate the confounding effect of albumin levels on the calculated apparent CL of VPA from the serum total concentrations (Css) in elderly epileptic patients. Consequently, the normalized total VPA concentrations (Css_N_) for a 42 g/L serum albumin concentration, which corresponds to the percentile 50 of the reference range for individuals between 25 and 55 years of age (13), were estimated as previously described (14), and the corresponding normalized apparent clearances (CL_N_) were calculated.

## Patients and methods

102 epileptic outpatients (53 males and 49 females) with a mean age (± SEM) of 40.2 ± 2.3 years (range 6–94 years) and orally treated with VPA in monotherapy were studied. None of them took drugs known to influence VPA–protein binding. In all cases the daily VPA administration was carried out in multiple doses with the same dosing interval. Blood samples were taken before breakfast and the morning dose, which had not been modified for at least 3 months prior to the study, and therefore the serum levels of VPA correspond to the trough steady-state concentrations (Css). This study was carried out according to the good practice rules for investigation in humans of the Conselleria de Sanidade (Regional Ministry of Health) of the Xunta de Galicia, Spain.

Serum VPA total concentrations were determined in a Dimension^®^ Xpand analyzer using reagents from Siemens Health Care Inc. (Newark, DE, USA). In cases with albumin concentrations lower or greater than 42 g/L (609 μmol/L), total VPA Css levels were normalized using the expression (14): Css_N_ = αCss/6.5, where 6.5 corresponds to the VPA unbound fraction for albuminemia of 42 g/L, and α is the estimated unbound fraction for a particular albumin concentration using the hyperbolic equation of Parent et al. (15): α (%) = Ae^-BX^, where X corresponds to albumin concentration (μmol/L), and the constants A = 130.69 and B = 4.96 × 10^-3^. For experimental total Css concentrations of VPA greater than 75 μg/mL, the normalized Css_N_ values were corrected as previously described (14). The VPA apparent CL or CL_N_ values were, respectively, calculated from the serum VPA Css or Css_N_ concentrations using a conventional pharmacokinetic procedure (12): CL (or CL_N_) = [(dose/dosing interval)/Css (or Css_N_)]. As in previous studies (9,10), trough rather than average VPA concentrations were used, and consequently the reported CL and CL_N_ represent overestimates of actual values.

Serum activities of alanine aminotransferase (ALT), aspartate aminotransferase (AST), and gamma-glutamyl transferase (GGT) and concentrations of albumin, bilirubin, and creatinine were determined in an Advia 2400 Chemistry System (Siemens Health Care Diagnostics Inc.). The platelet count was measured in blood samples collected 2–3 hours beforehand in K_3_EDTA anticoagulated tubes using an Advia 2120 Hematology System (Siemens Health Care Diagnostics Inc.). The liver fibrosis AST to platelet ratio index (APRI) was calculated in accordance with Wai et al. (16): APRI = [AST:URL/platelet count (10^9^/L)] × 100, where URL corresponds to the AST upper reference limits for men and women. Glomerular filtration rate (GFR) was estimated from serum creatinine using the Chronic Kidney Disease Epidemiology Collaboration (CKD-EPI) equation (17).

Statistical analysis of data was performed using the StatGraphics package, and the Kolmogorov–Smirnov test was applied to check for normality. The Pearson correlation coefficient and Student’s *t*-test were used when the data had a Gaussian distribution. Otherwise, the Spearman correlation coefficient and Mann–Whitney *U* test were used. In accordance with the consensus criteria for determination of drugs and their metabolites in biological samples (18), the accepted level for accuracy is a deviation of no more than 15% from the nominal value. Results were expressed as mean ± SEM (median).

## Results

There was a negative correlation between the serum albumin concentration and age (*r* = –0.462, *P* < 0.001), with patients older than 65 years of age presenting a significant decrease of the albumin concentration (*P* < 0.001) (Table I). In a majority of patients (*n* = 97), estimated GFR values were higher than 60 mL/min/1.73 m^2^. In all cases the levels of bilirubin were lower than the upper limit of reference (data not shown).

**Table I.** Pharmacokinetic and biochemical variables according to age in epileptic patients treated in monotherapy with valproic acid.

	≤19 years	20–64 years	≥65 years
*n* (males/females)	24 (14/10)	57 (29/38)	21 (10/11)
Age (years)	13.7 ± 0.9 (15.0)	37.9 ± 1.6 (38.0)	76.9 ± 1.7 (76.0)
VPA dose (mg/day)	836.7 ± 91.0 (800.0)***	1208.8 ± 52.5 (1200.0)	1066.7 ± 70.3 (1000.0)
VPA Css (μg/mL)	66.4 ± 4.6 (67.5)	67.9 ± 2.3 (68.9)	60.4 ± 4.3 (54.4)
VPA CL (mL/min)	9.2 ± 0.8 (8.8)***	12.7 ± 0.6 (11.7)	13.9 ± 2.0 (11.45)
VPA α (%)	5.87 ± 0.13 (5.6)*	6.59 ± 0.21 (6.2)	9.92 ± 1.09 (8.5)****
VPA Css_N_ (μg/mL)	76.2 ± 9.7 (59.0)	96.7 ± 10.4 (67.9)	145.3 ± 27.7 (71.1)
VPA CL_N_ (mL/min)	9.3 ± 0.9 (10.0)	11.4 ± 0.8 (11.1)	8.1 ± 0.9 (6.7)**
Albumin (g/L)	44.2 ± 1.0 (45.0)*	43.1 ± 1.0 (43.0)	37.0 ± 1.1 (38.0)****
AST (U/L)	14.7 ± 0.7 (14.5)	15.0 ± 1.1 (14.0)	17.6 ± 3.4 (13.0)
ALT (U/L)	14.9 ± 2.3 (12.0)*	19.9 ± 2.0 (15.0)	17.3 ± 3.7 (13.0)
GGT (U/L)	12.7 ± 3.1 (8.5)	17.0 ± 1.9 (12.0)	25.7 ± 6.8 (13.0)
APRI score	0.28 ± 0.02 (0.24)	0.33 ± 0.03 (0.28)	0.48 ± 0.12 (0.31)
AST/ALT ratio	1.2 ± 0.1 (1.2)****	0.9 ± 0.1 (0.7)	1.1 ± 0.1 (1.0)***
GFR (mL/min/1.73 m^2^)	144.0 ± 3.1 (144.2)****	102.4 ± 2.6 (102.8)	73.2 ± 4.0 (79.2)****

Statistical significance against non-elderly adult patients (20–64 years): *P < 0.05, **P < 0.01, ***P < 0.005, ****P < 0.001. Results are expressed as mean ± SEM (median).

**Figure 1.**
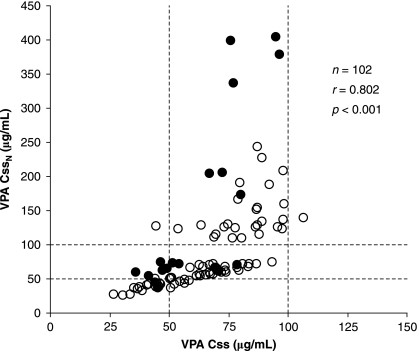
Relationship between total (Css) and albumin normalized total (Css_N_) concentrations of valproic acid in epileptic patients of <65 (○) and ≥65 (•) years of age. The dashed lines correspond to the limits of the valproic acid therapeutic range (50–100 μg/mL).

There was a relationship between total Css and normalized total Css_N_ VPA serum levels (Figure 1). For total trough VPA serum concentrations a therapeutic range of 50–100 μg/mL has been widely accepted (19), and a considerable proportion of cases presenting therapeutic Css levels, after its normalization by albumin concentration, present supratherapeutic Css_N_ levels, with a modest concordance of 53% in the classification of Css and Css_N_ levels as subtherapeutic, therapeutic, or supratherapeutic.

The different pharmacokinetic variables assayed were similar in patients aged between 20–40 and 41–64 years, and therefore these patients were included in one group of non-elderly adults with age between 20 and 64 years. Results obtained for the VPA daily doses, Css, Css_N_, CL, and CL_N_ in the studied patients grouped according to age are shown in Table I. The estimated VPA unbound fraction was significantly greater in the group of elderly patients. Significant differences for total VPA Css and Css_N_ concentrations between the different groups of patients were not obtained, and the difference between the medians of Css and Css_N_ was greater than 15% of median Css only in elderly patients. With respect to the VPA daily dose and apparent CL, these variables were significantly lower in patients aged ≤19 years (*P* < 0.005).

After normalization of serum VPA levels with the albumin concentration, the apparent CL_N_ was significantly decreased by around 40% in elderly patients (≥65 years). No significant correlation was found between the VPA normalized apparent CL_N_ and GFR (*r* = 0.074). The interindividual variation of the VPA apparent CL was significantly higher in patients aged ≥65 years than in the group of non-elderly adults (CV = 64% versus CV = 35%); however, the interindividual variation of the VPA normalized apparent CL_N_ was similar in both groups of patients (CV = 50% versus CV = 52%). A dichotomy of the data based on the patients’ sex did not reveal results with additional interest.

Calculation of APRI suggests a low likelihood of significant hepatic fibrosis in our VPA-treated patients, because in 84 cases (83%) the APRI score was ≤0.4, excluding the presence of liver fibrosis with a high level of confidence (16,20), and the APRI score was only higher than 1.5 in two cases, in which significant liver fibrosis may be supposed (16). In the group of patients with APRI score >0.4 the VPA apparent CL_N_ was significantly lower (*P* < 0.05) than in the group with APRI score ≤0.4 [7.3 ± 1.1 mL/min (6.5 mL/min) versus 10.5 ± 0.5 mL/min (10.8 mL/min)]. Likewise, significant negative correlations were found between the VPA apparent CL_N_ and the APRI (*r* = –0.258, *P* < 0.005), and AST/ALT ratios (*r* = –0.387, *P* < 0.001). The apparent CL_N_ was also significantly correlated with serum ALT (*r* = 0.280, *P* < 0.01) and GGT (*r* = 0.211, *P* < 0.05) activities, but not with AST activity (*r* = 0.076).

## Discussion

Age-related pharmacokinetic changes are due to multiple factors and present a high interindividual variability. Gastrointestinal absorption is not generally altered in elderly; nevertheless, decreased plasma protein binding, increased volume of distribution, reduced efficiency of drug-metabolizing enzymes, and diminished drug renal clearance may lead to a decreased elimination half-life of several drugs in older patients (11,22).

In accordance with previously published reports (7–10), the apparent CL of VPA calculated from the total Css concentrations was analogous in elderly and non-elderly adult patients (Table I); however, the serum concentration of albumin was significantly lower in the group with age ≥65 years than in the other groups that were considered (Table I), and therefore an increase of the VPA unbound fraction should be assumed in these elderly patients, as previously described (5,11,21,22). Normalization of total VPA Css concentrations permit estimation of the total Css_N_ levels (the unbound concentrations remain unchanged) that would be expected if the serum albumin concentration in all patients was 42 g/L (14) and consequently a correction of the confounding effect of serum albumin concentrations on the calculated apparent CL from total VPA levels.

At present, the clinical usefulness of measurements of VPA unbound concentrations is a debated subject (19,23); however, decreases in VPA protein binding are followed by reductions in total serum concentrations, whereas unbound concentrations are unchanged, and these reduced total concentrations may be misinterpreted as reflecting an inadequate dosage (24). As previously described for total and unbound VPA serum concentrations (25,26), a non-linear, hyperbolic relationship was found between total Css and total normalized Css_N_ concentrations (Figure 1). Similarly, a poor concordance of 53% was obtained in the classification of total Css and total normalized Css_N_ serum VPA levels as subtherapeutic, therapeutic, or supratherapeutic. The diagnostic efficiency of a laboratory test is the percentage of all results that are true, and as a general rule a test is probably not worth doing if its efficiency is less than 80% (27). Consequently the results indicated above suggest an unacceptable diagnostic efficiency of the total Css concentrations for therapeutic VPA monitoring in our group of epileptic out-patients. In a significant number of cases (around 47%) the determination of unbound VPA concentrations, or at least estimation of total normalized Css_N_ levels, would be clinically useful. In several cases, with VPA Css concentrations in the therapeutic range, the estimated normalized Css_N_ levels were significantly supratherapeutic (>200 μg/mL), explaining the hyper-sedation and dysarthria observed in these patients.

After correction of the masking effect of the albumin concentration, the apparent VPA CL_N_ calculated from the total normalized Css_N_ levels was significantly lower in the group of elderly patients than in the non-elderly adult patients group (*P* < 0.01). The VPA CL_N_ median decrease of about 40% in old age (Table I) is analogous to the difference previously calculated on apparent CL of unbound VPA (6,28). Also, after normalization of the total VPA Css concentrations, the interindividual variation of the normalized apparent CL_N_ calculated from Css_N_ was similar in all groups of patients, demonstrating that the higher variability of the apparent CL in the group of elderly patients is mainly due to their lower serum albumin concentrations (Table I).

A high prevalence of ultrasound-diagnosed non-alcoholic fatty liver disease has been previously demonstrated in adolescents (29) and adults (30,31) chronically treated with VPA. Fatty liver disease ranges from simple liver steatosis to steatohepatitis with necroinflammation and liver fibrosis, which can progress to cryptogenic cirrhosis, and significant changes occur in hepatic drug-metabolizing cytochrome P450 (CYP) enzyme families during these progressive stages (32). Although the principal pathways of VPA metabolism are glucuronidation and β-oxidation, and CYPs pathways account for less than 10% of the dose (33), functional polymorphisms of several cytochrome oxidase isoenzymes may explain part of the inter-individual variability in VPA pharmacokinetics (34). In accordance with previously published results (35), data indicated above for APRI suggest a low likelihood of significant liver fibrosis in epileptic patients treated with VPA in monotherapy, even in elderly subjects (Table I); however, significant negative correlations were obtained between the apparent CL_N_ of VPA and the APRI score and AST/ALT ratio.

Induction of CYPs and GGT by enzyme-inducing anticonvulsant drugs is widely documented and in epileptic patients treated with VPA in monotherapy and polytherapy with carbamazepine, phenytoin, or phenobarbital; a significant correlation has been previously reported between the apparent CL of VPA and ALT and GGT serum activities (33). In our patients that were treated with VPA in monotherapy, significant positive correlations were also found between the apparent CL_N_ of VPA and serum GGT and ALT. VPA is not an enzyme-inducing agent, and in these patients GGT and ALT activities may be associated with over-weight and fat accumulation in the liver (33,35). Although in simple steatosis the function of several liver CYPs isoenzymes may be impaired, the activity of phase II conjugation enzymes, such as UDP-glucuronyltransferase, would be enhanced (36), with the consequent increase of VPA biotransformation and elimination.

The main limitation of our study is that the VPA free fraction was not experimentally determined. Endogenous substances such as free fatty acids, uremic compounds, and bilirubin may displace VPA from its albumin binding sites, increasing the unbound fraction and leading to an underestimation of the normalized Css_N_ values. Free fatty acids commonly increase after fasting states, and in our study blood samples were taken before breakfast; in none of our patients was a situation of renal failure or jaundice observed. Although theoretical estimation of normalized Css_N_ concentrations offers approximate results (14), this pharmacokinetic approach permits evaluation of the confounding effect of the albumin concentration on the apparent CL of VPA in old age.

In conclusion, total serum levels of VPA should be considered with caution, principally in older patients, in which determination of the drug unbound concentrations, or at least normalized total drug levels for 42 g/L albumin concentration, may be clinically useful. Normalization of total VPA levels permits estimation of a decrease around 40% for the apparent CL in elderly patients.
